# Generation of Multi-Transgenic Pigs Using PiggyBac Transposons Co-expressing Pectinase, Xylanase, Cellulase, β-1.3-1.4-Glucanase and Phytase

**DOI:** 10.3389/fgene.2020.597841

**Published:** 2020-11-30

**Authors:** Haoqiang Wang, Guoling Li, Cuili Zhong, Jianxin Mo, Yue Sun, Junsong Shi, Rong Zhou, Zicong Li, Zhenfang Wu, Dewu Liu, Xianwei Zhang

**Affiliations:** ^1^National Engineering Research Center for Breeding Swine Industry, College of Animal Science, South China Agricultural University, Guangzhou, China; ^2^Wens Foodstuff Group Co., Ltd., Yunfu, China

**Keywords:** transgenic pigs, digestive enzymes, salivary gland, polycistronic, PiggyBac

## Abstract

The current challenges facing the pork industry are to maximize feed efficiency and minimize fecal emissions. Unlike ruminants, pigs lack several digestive enzymes such as pectinase, xylanase, cellulase, β-1.3-1.4-glucanase, and phytase which are essential to hydrolyze the cell walls of grains to release endocellular nutrients into their digestive tracts. Herein, we synthesized multiple cellulase and pectinase genes derived from lower organisms and then codon-optimized these genes to be expressed in pigs. These genes were then cloned into our previously optimized *XynB* (xylanase)- *EsAPPA* (phytase) bicistronic construct. We then successfully generated transgenic pigs that expressed the four enzymes [*Pg7fn* (pectinase), *XynB* (xylanase), *EsAPPA* (phytase), and *TeEGI* (cellulase and β-glucanase)] using somatic cell cloning. The expression of these genes was parotid gland specific. Enzymatic assays using the saliva of these founders demonstrated high levels of phytase (2.0∼3.4 U/mL) and xylanase (0.25∼0.42 U/mL) activities, but low levels of pectinase (0.06∼0.08 U/mL) activity. These multi-transgenic pigs are expected to contribute to enhance feed utilization and reduce environmental impact.

## Introduction

Ineffective digestion in pigs causes excess nutrients into be released to the environment. This results in soil salinity and potential pollution to water and air (Shirali et al., 2012). Domestic pigs mainly feed on common cereal grains, oil seed meals and their by-products. These contain various anti-nutritional factors such as non-starch polysaccharides and phytic acid (Gilani et al., 2005; Bohn et al., 2008). These anti-nutritional factors have an obvious effect on the digestion and absorption of nutrients. It hinders the contact of endogenous digestive enzymes with chyme and hence slows down the nutritional diffusion rate into the intestines (Shirali et al., 2012). As a consequence, undigested nutrients containing large amounts of inorganic nitrogen and phosphorus are excreted by pigs. This subsequently stimulates the growth of algae and other aquatic plants when they contaminate rivers and streams. This in turn enhances microbial proliferation to ultimately contribute to air pollution.

Several dietary manipulation strategies have been employed to reduce fecal output and nutrient excretion in swine. The most widely practiced strategy is to introduce phytate or non-starch polysaccharides, which are degrading enzymes in feed formula. These could effectively decrease nitrogen and/or phosphorus emissions and hence reduce environmental impact. However, various factors affect the catalytic activity of these microbial enzymes, such as feed processing and storage, feed components, pH, minerals, and temperature. Recently, genetically engineered pigs that express specific or multiple digestive enzymatic genes have provided an alternative strategy to replace dietary enzyme supplementation in the feed. Recently study demonstrated that transgenic pigs that produce salivary phytase had less than 75% of fecal phosphorus. In addition, these pigs required almost no inorganic phosphate supplementation for normal growth compared to non-transgenic pigs (Golovan et al., 2001). In our previous study, we established transgenic pigs that simultaneously expressed three microbial enzymes, β-glucanase, xylanase, and phytase in their salivary glands. These pigs had significantly enhanced growth and reduced fecal nitrogen and phosphorus levels (Zhang et al., 2018).

Cell wall of cereals is mainly composed of cellulose, hemicellulose, and pectin. These components cannot be digested by pigs, which leads to a part of energy loss. Therefore, the expression of pectinase and cellulase in the digestive tract of pigs seems to have crucial and potential value. Among them, pectinase can separate the cellulose molecules wrapped by pectin and reduce the feed viscosity, which increases absorption and release of nutrients, either by hydrolyzing non-biodegradable fibers or by liberating nutrients blocked by these fibers and reduces fecal output (Hoondal et al., 2002). Additional, cellulose and hemicellulose digested by cellulase become monosaccharides or oligosaccharides, which are then absorbed by the digestive tract. The expression of these enzymes in porcine saliva is more convenient to detect than in other places, such as the pancreas (Lin et al., 2015). In this study, we isolated and characterized several novel digestive enzyme genes, and then generated transgenic pigs that expressed these multiple enzymes. These included pectinase, xylanase, cellulase, β-1.3-1.4-glucanase, and phytase. These genes were expressed using a salivary gland promoter. These transgenic pigs had no adverse reactions and had better feed digestion compared to non-transgenic pigs.

## Materials and Methods

### Ethics Statement

All experimental animal protocols were in accordance with the care and use of laboratory animals issued by the Ministry of Science and Technology of China. The use of animal experiments was approved by the Institutional Animal Care and Use Committee of South China Agricultural University.

### Plasmid Construction

Three pectinase genes, *PgaA* (*Aspergillus niger* JL-15) (Liu et al., 2014), *Pg7fn* (*Thielavia arenaria* XZ7) (Tu et al., 2014) and *PGI* (*chaetomium* sp.) (Tu et al., 2013); one xylanase *gene XynB* (*Aspergillus niger*) (Deng et al., 2006; Zhang et al., 2018), one phytase gene *EsAPPA* (*Escherichia coli*) (Zhang et al., 2018) and six cellulase and β-glucanase genes (respectively), *cel5B* (*Gloeophyllum trabeum*) (Kim et al., 2012), *egII* (*Pichia pastoris*) (Akbarzadeh et al., 2014), *AG-egaseI* (*Apriona germari*) (Lee et al., 2004), *TeEGI* (*Teleogryllus emma*) (Kim et al., 2008), *cel9* (*Clostridium phytofermentans*) (Zhang et al., 2010) and *Bh-egaseI* (*Batocera horsfieldi*) (Mei et al., 2016) were synthesized by Genscript (Nanjing, China) that were pig codon-optimized. They were then cloned into pcDNA3.1(+). *Pg7fn*, *XynB*, *EsAPPA* and *TeEGI* genes were then head-to-tail ligated using E2A, P2A, and T2A linkers. The ligated construct was named *PXAT*. *PXAT* was then inserted into pcDNA3.1(+) and enzymatic activity was then evaluated. *PXAT* was also inserted into the tissue-specific vector (pPB-mPSP-loxp-neoEGFP-loxp) to generate the final transgene construct (mPSP-PXAT). The primer sets used for cloning are listed in Supplementary Table 1.

### Cell Culture and Transfection

The PK-15 cell line (ATCC CCL-33) and porcine fetal fibroblasts (PFFs) were cultured in DMEM (Thermo Fisher Scientific, Suwanee, GA, United States) supplemented with 10% fetal bovine serum (Thermo Fisher Scientific, Suwanee, GA, United States). To evaluate enzymatic activity, PK-15 were grown to 70% confluence, and then transfected using lipofectamine LTX reagent (Thermo Fisher Scientific, Suwanee, GA, United States). Sixty hours post-transfection, the culture supernatant was collected for enzymatic assays. For transgene cell line selection, PFFs were co-electroporated with a circular transposase plasmid (pCMV-hyPBase) and the circular mPSP-PXAT plasmid using the program A-033 on the Nucleofector 2b Device (Amaxa Biosystems/Lonza, Cologne, Germany). After cell attachment, 400 μg/ml G418 (Gibco) was added to the culture media for transfected cell selection. Clonal cells expressing green fluorescence were selected and identified using PCR and sequencing.

### Generation of Transgenic Pigs

The EGFP marker gene and neomycin resistant gene (neoR) were removed from transgenic cells using the Cre enzyme (Excellgen, Rockville, MD, United States) and then mixed multiple positive clones as nuclear donors for somatic cell nuclear transfer. Somatic cell nuclear transfer was described as previously studied (Zhang et al., 2018). The reconstructed embryos were transferred into recipient gilts, and piglets were naturally born after gestation. Afterward, genomic DNA from tail was extracted and sequenced using PCR (Supplementary Table 2). Additionally, mRNA was extracted from porcine tissue samples and reversed transcribed to cDNA to be used as the template for reverse transcriptase PCR (RT-PCR) and quantitative Real-time PCR (qPCR) (primers used are listed in Supplementary Table 3). Relative qPCR and absolute qPCR were used to identify mRNA expression levels and copy number in transgenic pigs, respectively.

### Southern and Western Blot Analysis

Genomic DNA was digested with restriction enzymes *Kpn* I or *Eoc47* III, and then run on an 0.8% agarose gel. The digested fragments were then transferred to a nylon membrane. The membrane was hybridized using digoxigenin-labeled DNA probes (Supplementary Table 2) for *mPSP* based on the DIG-High Prime DNA Labeling and Detection Starter Kit II protocol (Roche, Mannheim, Germany). For western blotting, saliva was collected and then ultra-filtrated using a centrifugal filter (Millipore, MA, United States). Total protein from saliva was then electrophoresed on an SDS polyacrylamide gel and subsequently transferred to a polyvinylidene fluoride membrane (Millipore, MA, United States). The membranes were incubated overnight at 4°C with primary rabbit polyclonal antibodies (Supplementary Table 4) against XynB or TeEGI (purchased from Genscript, Nanjing, China). The salivary amylase antibody (ab34797, Abcam) was used to confirm equal protein loading and the dilution ratio was 1: 1000. Membranes were then washed and incubated with a secondary IgG antibody. Bands were visualized using the UVP software.

### Enzymatic Activity Assay

Cell culture supernatants, porcine saliva and rumen fluid of cattle were centrifuged and used for enzymatic activity assay. Saliva collection has been described in previous study (Zhang et al., 2018). Pectinase, xylanase, β-glucanase and cellulase activities were assayed using 1% (w/v) polygalacturonic acid (and 55∼70% esterified pectin, >85% esterified pectin), 1% (w/v) xylan, 0.8% (w/v) β-d-glucan, and 1% (w/v) sodium carboxymethyl cellulose as the substrates, respectively. Reducing sugar content was measured using the 3,5-dinitrosalicylic acid (DNS) method (Kim et al., 2012; Tu et al., 2013). One unit of enzymatic activity was defined as the rate at which 1 μmol of reducing sugar was released per minute. Phytase activity in saliva was measured as previously described (Zhang et al., 2018).

The optimal pH of these proteins was determined at 39.5°C for 30 min in buffers of pH 1.0∼8.0. The buffers used were 0.2 M potassium chloride (KCl)- hydrochloric acid (HCl) for pH 1.0, 0.2 M glycine-HCl for pH 2.0∼3.0, and 0.2 M citric acid-disodium hydrogen phosphate (Na_2_HPO_4_) for pH 4.0∼8.0. All protein tolerance tests were measured after buffer treatment for 2 h under optimal conditions (optimal pH, 39.5°C and 30 min).

### Feeding Management

Transgenic pigs and wild-type littermates were fed on the same diet (Supplementary Table 5). They were raised in the same pens fitted with MK3 FIRE feeders (FIRE, Osborne Industries Inc., Osborne, KS, United States). Individual daily feed intake and body weight were recorded when the pigs accessed the FIRE feeders. All pigs had free access to feed and drinking water throughout the growth phase. Blood was collected in a sterile manner at 90 days of age. Serum biochemical parameters of growing-finishing pigs were determined using a Hitachi 7020 full-automatic biochemical analyzer (Japan).

### Statistical Analysis

Data were analyzed using the IBM SPSS Statistics 20 (IBM SPSS, Chicago, IL, United States) or SAS 9.4 (SAS Inst. Inc., Cary, NC, United States). For enzymatic activities analysis and relative gene expression, one-way ANOVA or *t*-test was used. For serum biochemical data, unpaired *t*-test (two-tailed) was used. For growth performance, a total of 3 F1 transgenic pigs (1 boar, 2 gilts) and 6 wild-type littermates (3 boars, 3 gilts) were test. When it comes to statistics, multivariate analysis of variance (MANOVA) was performed using the GLM procedure, with sex and initial weight used as the covariate (Zhang et al., 2018). Data were expressed as mean ± SEM. *P* < 0.05 considered statistically significant.

## Results

### Characterization of the Three Pectinase Genes Expressed in PK-15 Cells

**FIGURE 1:**
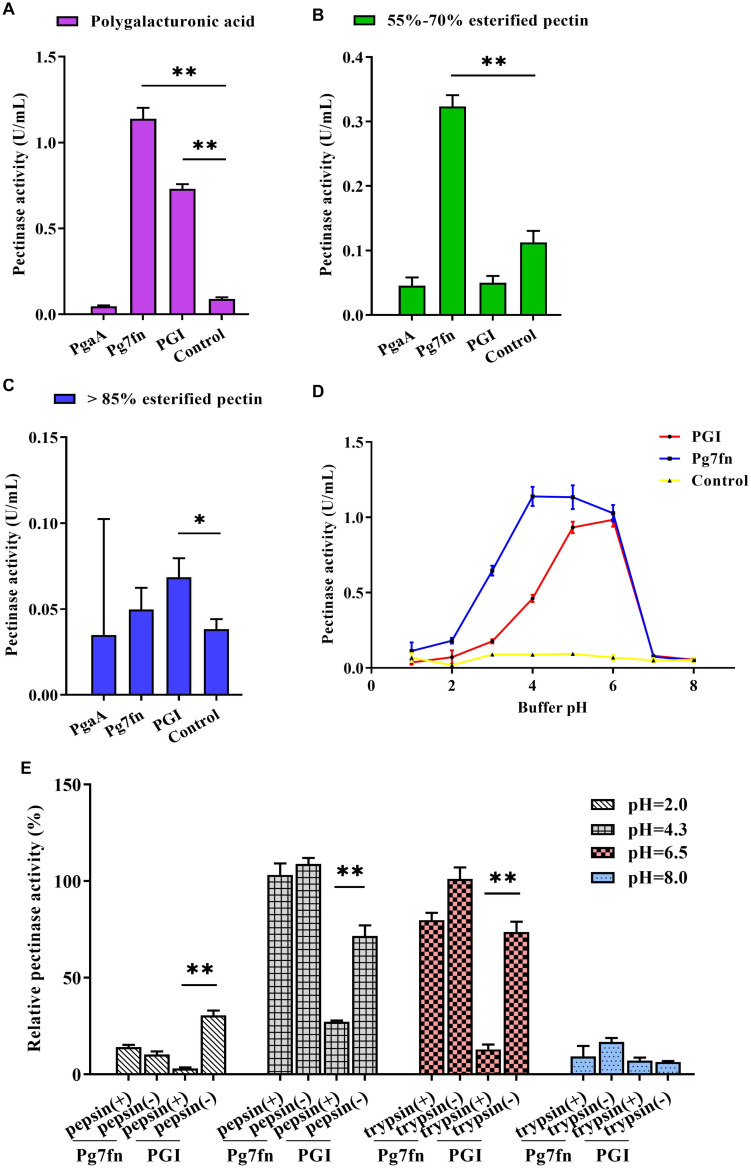
Characterization of the three pectinase genes expressed in PK-15 cells. Pectinase activities of *PgaA, Pg7fn*, and *PGI* were evaluated using 1% **(A)** polygalacturonic acid, **(B)** 55∼70% esterified pectin, and **(C)** >85% esterified pectin as substrates at pH 4.5, respectively. **(D)** Pectinase activities of *Pg7fn* and *PGI* at different pH levels (1.0∼8.0). **(E)** *Pg7fn* and *PGI* were incubated with different pepsin and trypsin pH solutions at 39.5°C for 2 h. Control represents the pcDNA 3.1(+) vector. Data were shown as mean ± SEM, *n* = 3 (one-way ANOVA). ^∗^*P* < 0.05, ^∗∗^*P* < 0.01.

Based on a previous study, we initially selected three pectinase genes *Pg7fn*, *PgaA*, and *PGI* for our studies. Enzymatic activity assays demonstrated that *Pg7fn* had the highest pectinase activity toward 1% polygalacturonic acid and 55∼70% for esterified pectin when used as the substrates, respectively. *PGI* had the second highest pectinase activity toward 1% polygalacturonic acid. However, the activities of *Pg7fn*, *PgaA*, and *PGI* were lower than 0.1 U/mL for >85% esterified pectin (Figures 1A–C). We selected *Pg7fn* and *PGI* to determine their optimal pH in 1% polygalacturonic acid. The enzymatic activity of *Pg7fn* increased with pH between 1.0 and 4.0 and reached the highest pectinase activity at pH 4.0, at approximately 1.15 U/mL. The high enzymatic activity was stable at pH 4.0∼6.0, and then decreased significantly after pH 6.0. *PGI* showed a similar trend as *Pg7fn*, but reached its highest enzymatic activity at pH 6.0 (Figure 1D). The relative pectinase activities of *Pg7fn* and *PGI* remained at least 56.8 and 46.8% during the stationary phase, respectively. To simulate the pig’s digestive tract, we treated *Pg7fn* and *PGI* at 39.5°C for 2 h with different pepsin and trypsin pH solutions. The results indicated that pectinase activity of *PGI* was significantly decreased after pepsin or pH 6.5 trypsin treatment (Figure 1E). However, *Pg7fn* was not affected by treatment with pepsin and trypsin. Hence, *Pg7fn* was selected as the candidate gene.

### Characterization of the Six Cellulase Genes Expressed in PK-15 Cells

**FIGURE 2:**
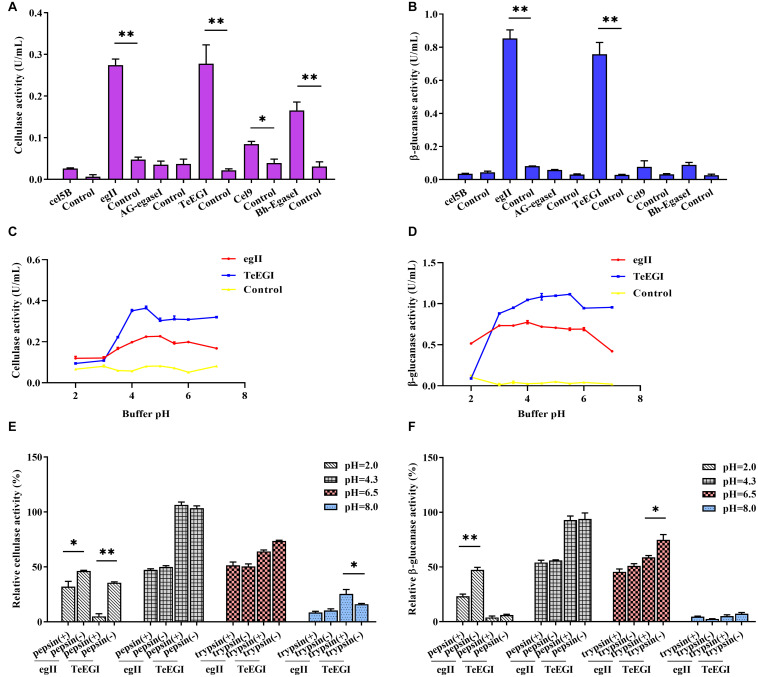
Characterization of six cellulase genes expressed in PK-15 cells. **(A)** Cellulase and **(B)** β-glucanase activities of *cel5B*, *egII*, *AG-egaseI*, *TeEGI*, *cel9*, and *Bh-egaseI* were evaluated at suitable pH conditions. **(C)** Cellulase activities of *egII* and *TeEGI* at different pH levels (2.0∼7.0). **(D)** β-glucanase activities of *egII* and *TeEGI* at different pH levels (2.0∼7.0). **(E)** Cellulase and **(F)** β-glucanase activities of *egII and TeEGI* were measured following incubation with different pepsin and trypsin pH solutions. Control represents the pcDNA3.1(+) vector. Data were shown as mean ± SEM, *n* = 3 (*t*-test). ^∗^*P* < 0.05 or ^∗∗^*P* < 0.01.

We selected six endo-β-1,4-endoglucanase genes (*cel5B*, *egII*, *AG-egaseI*, *TeEGI*, *cel9*, and *Bh-egaseI*) to measure cellulase and β-glucanase activities at various pH conditions. *egII* and *TeEGI* cellulase activities were significantly higher (0.27 and 0.28 U/mL, respectively) compared to the other genes for 1% sodium carboxymethyl cellulose (Figure 2A). Furthermore, β-glucanase activities of *egII* and *TeEGI* were approximately 0.76 and 0.86 U/mL for 0.8% β-D-glucan as substrate, respectively. The other genes had activities of less than 0.09 U/mL (Figure 2B). To further determine the enzymatic characteristics of *egII* and *TeEGI*, we optimized the pH levels of the reaction buffer. We found that *TeEGI* had the highest cellulase activity at pH 4.5 and had higher residual activity after treatment with pH 3.5∼7.0 (Figure 2C). *egII* had similar trends, however, the optimal pH was 5.0. The β-glucanase activity of *TeEGI* was greater than 0.88 U/mL at pH 3.0∼7.0 and reached a maximum of 1.11 U/mL at pH 5.5 (Figure 2D). Compared to *TeEGI*, the highest β-glucanase activity of *egII* was 0.77 U/mL and had a residual activity of greater than 50% between pH 2.0∼7.0. We then investigated whether *egII* and *TeEGI* would have high enzymatic activity in different pepsin and trypsin pH buffers. The results indicated that *TeEGI* was resistant to pepsin and trypsin digestion, but *egII* β-glucanase and cellulase were significantly inhibited at pH 2.0 pepsin buffer (Figures 2E,F). Hence, we selected *TeEGI* as the candidate cellulase and β-glucanase gene.

### Enzymatic Activity Between Polycistronic and Monomeric Constructs

**FIGURE 3:**
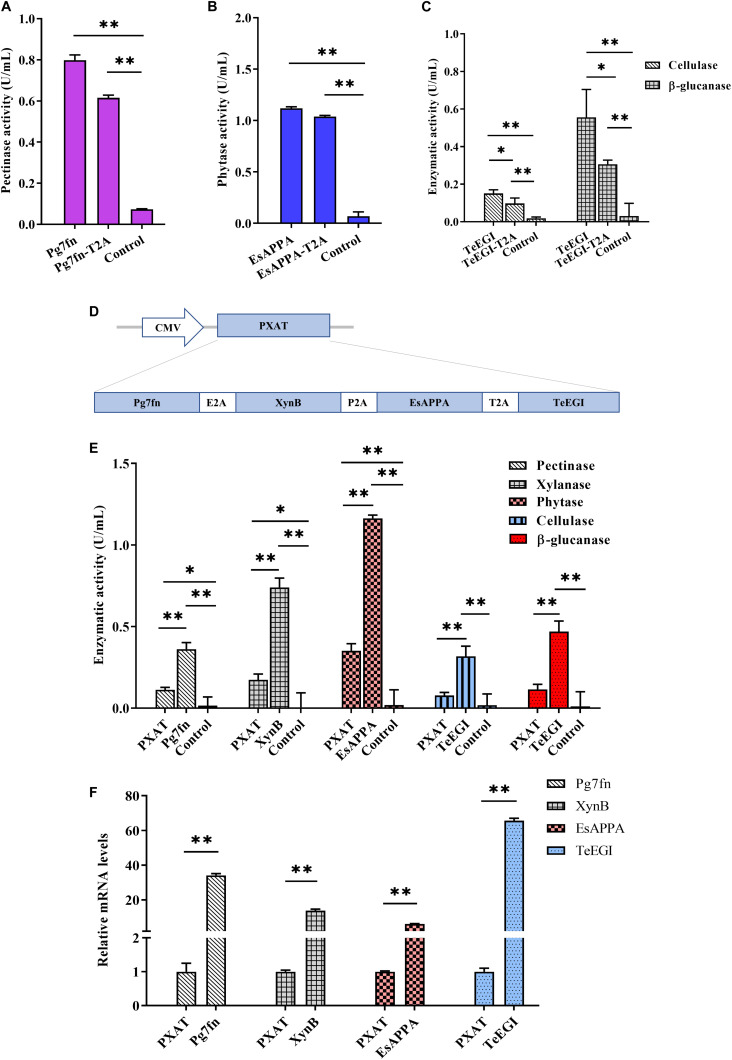
Enzymatic activities between the polycistronic and single gene vector constructs. The effect of 2A linker peptide on **(A)** pectinase, **(B)** phytase, **(C)** cellulase, and β-glucanase activity. **(D)** Schematic of the *PXAT* vector. **(E)** Enzymatic activities between PXAT and its corresponding protein expressed by single gene constructs. **(F)** Relative mRNA expression levels between genes expressed with PXAT and single gene constructs. Control represents the pcDNA3.1(+) vector. Data were shown as mean ± SEM, *n* = 3 (one-way ANOVA). ^∗^*P* < 0.05 or ^∗∗^*P* < 0.01.

To assess the polycistronic positions of the four genes (*Pg7fn*, *TeEGI*, *EsAPPA*, and *XynB*), we initially included the 2A linker at the end of each corresponding gene. Previous studies had demonstrated that the XynB protein with the P2A residue at the C-terminus still had high xylanase activity in porcine saliva (Zhang et al., 2018). Our results demonstrated that the enzymatic activities of Pg7fn and EsAPPA with 2A residue also kept high relative activity (>77 and >92%, respectively) (Figures 3A,B). However, cellulase and β-glucanase activities of *TeEGI* with T2A were significantly reduced to 64.8 and 55.1%, respectively (Figure 3C). We fused *Pg7fn*, *XynB*, *EsAPPA*, and *TeEGI* genes head to tail with E2A, P2A, and T2A linkers, and named the final construct *PXAT* (Figure 3D). *PXAT* was then ligated into pcDNA3.1(+) to evaluate enzymatic activity. The results showed that using *PXAT*, the pectinase, xylanase, phytase, cellulase, and β-glucanase activities were significantly reduced to 31.0, 23.5, 30.2, 24.5, and 24.4%, respectively, compared to constructs expressing a single gene (Figure 3E). RT-PCR further confirmed that the four co-expressed genes had lower mRNA levels compared to single gene constructs (Figure 3F).

### Generation and Identification of Transgenic Pigs

**FIGURE 4:**
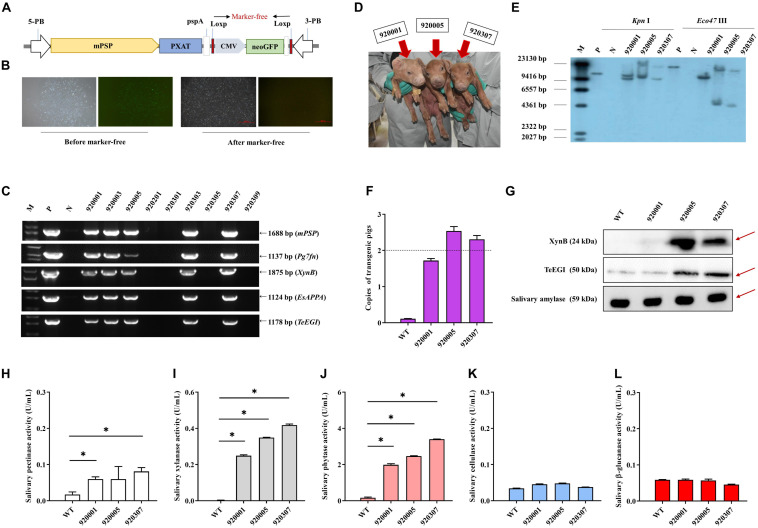
Generation and identification of the transgenic pigs. **(A)** Schematic of the transgenic plasmid mPSP-PXAT. The mPSP-PXAT consisted of the mouse parotid secretory protein (mPSP) promoter, loxp system with the neo-EGFP marker protein, and a PiggyBac transposon. **(B)** EGFP was deleted using Cre recombinase prior to somatic cell nuclear transfer. **(C)** Genomic identification of transgenic piglets using PCR and gel electrophoresis. **(D)** Transgenic piglets at 2-week-old. **(E)** Southern blot analysis of transgene integration in transgenic piglets. Genomic DNA was digested using *Kpn* I and *Eco47* III endonucleases. **(F)** Copy number determination in transgenic piglets by absolute quantification. **(G)** Western blotting analysis of XynB and TeEGI protein expression. Salivary amylase was used as a protein reference. **(H)** Salivary pectinase, **(I)** xylanase, **(J)** phytase, **(K)** cellulase, and **(L)** β-glucanase expression at 4 months. M is the DNA marker, P indicates mPSP-PXAT plasmid; N and WT represent wild-type pigs. Data were shown as mean ± SEM (one-way ANOVA). ^∗^*P* < 0.05.

*PXAT* was also inserted into the tissue-specific vector pPB-mPSP-loxp-neoEGFP- loxp to form the final transgene construct (mPSP-PXAT) (Figure 4A). The mPSP-PXAT contained the mouse parotid secretory protein (mPSP) promoter, loxp flanking the neo-EGFP marker genes, and the left and right ends of the PiggyBac elements. For transgene cell line selection, PFFs were co-electroporated and G418 was used for selection. The EGFP marker gene was deleted in clonal cells using Cre enzyme prior to somatic cell nuclear transfer (Figure 4B). A total of two cell lines were pooled and used as nuclear donors. We transferred a total of 2,096 reconstructed embryos into 10 recipient gilts. Four recipients became pregnant and delivered nine Duroc piglets, of which seven were alive and two were dead (Supplementary Table 6). PCR sequencing demonstrated that the five founders were positive for the transgene (Figure 4C), but only three of which were alive (Figure 4D). Southern blot and quantitative PCR demonstrated that three piglets carried two copies of the transgene (Figures 4E,F). A positive boar was euthanized, and tissue samples were collected to determine expression levels of transgenic mRNA at 10 months of age. The results showed that the four genes, i.e., *Pg7fn*, *XynB*, *EsAPPA*, and *TeEGI* were highly expressed in the parotid gland, had low expression in the sublingual and submandibular gland, and not expressed in other tissues (Supplementary Figures 1,2). Enzymatic activity assays showed that the saliva from three founders was positive for pectinase, xylanase, and phytase (0.06∼0.08, 0.24∼0.42, 1.9∼3.4 U/mL, respectively) (Figures 4H–L). Although we were unable to detect cellulase and β-glucanase activities, interestingly the western blotting analysis indicated that the TeEGI protein was expressed (Figure 4G). The F1 pigs (6 transgenic pigs and 6 wild-type littermates) were obtained from 920307 transgenic pig by mating with 2 wild-type gilts (Supplementary Figure 3). The results revealed that F1 pigs had pectinase, xylanase, and phytase activities, but no cellulase and β-glucanase activities (Supplementary Figure 4), which were consistent with the founders. We also measured serum biochemical markers in both F1 transgenic and wild-type pigs (Supplementary Table 7). The results showed that the phosphorus content of transgenic pigs (3.32 mM) was higher compared to wild-type pigs (2.79 mM), which revealed that PXAT pigs maybe promote phosphorus absorption in feed. The growth data of F1 transgenic pigs and wild-type littermates were measured (Supplementary Table 8), which shown that PXAT pigs had a tendency to improve growth performance (Supplementary Figure 5). Transgenic pigs had better feed conversion ratios compared to wild-type pigs fed the same diet and it took an average of 84 days for transgenic pigs to grow from 30 to 100 kg, whereas wild-type pigs required about 96 days. Compared with wild-type pigs, our transgenic pigs improved feed conversion efficiency by 10.94% and saved an average of 7.08% in feed costs per pig at the 30∼100 kg stage. The growth curve revealed that transgenic pigs grew faster than the littermates, mainly at the 50∼70 kg stage (Supplementary Figure 6).

## Discussion

Environmentally friendly transgenic pigs could efficiently improve the absorption of anti-nutritional factors, have enhanced growth, and emit lower levels of nitrogen and phosphorus into the environment (Zhang et al., 2018). Previous studies have demonstrated that salivary phytase and xylanase produced from transgenic pigs could effectively reduce phosphorus and nitrogen emissions (Golovan et al., 2001; Zhang et al., 2019). However, no studies to date have investigated cellulase or pectinase transgenic pigs. In this study, we initially selected three pectinase genes (*PgaA, Pg7fn*, and *PGI*) and six cellulase genes (*cel5B, egII, AG-egaseI, TeEGI, cel9*, and *Bh-egaseI*) based on previous studies. Our results demonstrated that *Pg7fn* and *TeEGI* had high enzymatic activities at different pH levels and maintained their stability in different pepsin and trypsin pH buffers. However, several genes had no detectable enzymatic activities. These genes were derived from microorganisms and insects, and the PK-15 cell line that was used to express these genes were unable to properly recapitulate the post-translation modifications needed for enzymatic activity. In addition, the polycistronic order of the four genes (*Pg7fn*, *XynB*, *EsAPPA*, and *TeEGI*) were constructed using the 2A linker at the end of each corresponding gene. In PK-15 cells, our result demonstrated that the target protein with 2A residue at the C-terminus significant reduced enzymatic activity, such as Pg7fn, EsAPPA, and TeEGI, in which, the activities of cellulase and β-glucanase (TeEGI) decline were most pronounced. It seemed that particular protein require special folding compared to the others. The 2A linker was derived from viruses, such as foot-and-mouth- disease virus (F2A), equine rhinitis A virus (E2A), thosea asigna virus (T2A), and porcine teschovirus-1 (P2A). When mRNA is translated, ribosomes jump from Gly to Pro in the 2A sequence. This results in the absence of a peptide bond between Gly and Pro. As a consequence, the upstream protein that is generated has a 17∼19 amino acid peptide that contains Gly at the C-terminus, while the downstream protein that is generated has a Pro residue at the N-terminus, which may affect the spatial folding of the protein. As mentioned previously, the incomplete cleavage of the 2A linker could reduce protein expression (Velychko et al., 2019). There is a parotid gland expression signal peptide in front of each gene, which seems to rule out the reason that the C-terminal protein stays on the endoplasmic reticulum due to the inability of 2A linker to completely cleave (De Felipe et al., 2010). In addition, some proteins may be unable to be completely synthesized due to incomplete translation. This may explain why some of the PXAT enzymatic activities were significantly reduced compared to proteins that were synthesized using the single-gene vector. Finally, the larger size of the PXAT construct may contribute to lower transfection efficiency compared to constructs having only a single gene (Kreiss et al., 1999).

We successfully generated three transgenic pigs expressing multiple digestive enzymatic genes using the PiggyBac transposon system. Although the transgenic pigs could efficiently express pectinase, xylanase, and phytase, we were unable to detect the enzymatic activity of cellulase and β-glucanase. Western blot analysis indicated that the TeEGI protein was expressed. Previous study suggested that different post-translational modification manners had an effect on protein function (Knorre et al., 2009). Thus, we suspect that TeEGI lacks cellulase activity, possibly due to post-translational modification that alters the folding or function of the protein. Additional, although cellulase was secreted in PK-15 cells, interaction between various cells in an individual could also affect protein function. The polyA tail plays a crucial role in transcription, translation and stabilization of mRNAs (Edmonds, 2002). In our previous work, we used the *bGH-pA* (bovine growth hormone polyadenylation signal) as a termination sequence (Zhang et al., 2018). But in this study, we firstly utilized an unconventional polyA (3′ UTR of parotid secretory protein as a termination sequence *pspA*) to evaluate its effect (Figure 4A). We inferred that *pspA* may affect the activity of cellulase and β-glucanase. Due to the unavailability of porcine parotid gland cell lines, we used the PK-15 cell line to express the four enzymatic genes driven by the CMV promoter. However, in animal models, multiple digestive enzymatic genes are driven by the parotid secretory protein promoter. Hence, the low enzymatic activity that was observed may be due to an incompatible promoter.

In previous study, transgenic founders expressed four genes for three digestive enzymes, which produced 0.34∼2.32 U/mL of β-glucanase, 0.40∼2.37 U/mL of xylanase, and 0.40∼5.7 U/mL of phytase in the saliva (Zhang et al., 2018). High β-glucanase was mainly supported by *bg17A* and *eg1314* genes not *TeEGI*. The transgenic founders in this study had a slightly lower xylanase activity (0.25∼0.42 U/mL) than the previous pigs. However, the pectinase (0.07∼0.83 U/mL) and xylanase (0.34∼1.20 U/mL) activities of the F1 generation have been improved, which may be related to age and individuals (Li et al., 2020). In addition, seasonal changes, eating environment and saliva collection methods may affect the protein content of saliva, thereby affecting the secretion of digestive enzymes (Sanchez et al., 2019). Thus, further exploration of porcine saliva secretion patterns in the future is essential for the study of saliva bioreactors. Moreover, with reference to previous research, we were able to predict that the genetically modified pigs in this study would have a weaker capacity to reduce emissions than the previous ones, mainly because their key enzyme (TeEGI) was inactivated in the salivary glands. Therefore, the establishment of a porcine parotid gland cell line is beneficial for the screening of polycistrons and the model preparation of saliva bioreactors.

The ability of ruminants to digest plant fibers is well known, and the average pH of their rumen tends to be neutral. However, as a monogastric animal, pigs have an average gastric pH of about 4.4 (Merchant et al., 2011), which causes most of the digestive enzymes derived from bacteria and other microorganisms to be inactivated in the acidic stomach. In order to have a clearer understanding of our transgenic pig’s ability to digest feed fibers, we measured their ability to digest anti-nutritional factors with bovine rumen. The results revealed that our transgenic pigs had better pectinase, xylanase and phytase activities than cattle, while the cellulase and glucanase activities of cattle were higher than those of our transgenic pigs (Supplementary Figure 7). The reason why the activities of pectinase and xylanase of cattle were lower than that of our genetically modified pigs was mainly related to the feed ingredients of cattle (Mendoza et al., 2014), which affected the microbial environment of cattle rumen (McCann et al., 2014). Glucanase or cellulase is an extremely important glycol- hydrolase for improving the digestibility of pig feed. Therefore, it may be more effective to screen out a variety of acid-resistant hydrolases or mutants, and even use acid-resistant molecular chaperones such as HdeA or HdeB on improving porcine digestibility and reducing environmental release in the future.

In summary, pork is a daily necessity of people, with an annual output exceeding 100 million tons. However, a significant amount of food was wasted due to porcine low feed utilization. Additional, nitrogen and phosphorus that can not be absorbed by pigs are discharged into the environment, which will increase the sewage treatment load and even cause water pollution. In this study, we successfully produced an animal model using somatic cell transfer. These transgenic pigs expressed, under the control of the parotid gland specific promoter, four enzymatic genes [*Pg7fn* (pectinase), *XynB* (xylanase), *EsAPPA* (phytase), and *TeEGI* (cellulase and β-glucanase)], although TeEGI was inactive. It offer a valuable experience for the global environmental concerns and the inefficient absorption of feed in livestock.

## Data Availability Statement

The original contributions presented in the study are included in the article/Supplementary Material, further inquiries can be directed to the corresponding authors.

## Ethics Statement

All experimental animal protocols were in accordance with the care and use of laboratory animals issued by the Ministry of Science and Technology of China. The use of animal experiments was approved by the Institutional Animal Care and Use Committee of South China Agricultural University.

## Author Contributions

HW, GL, and XZ wrote the manuscript. XZ, DL, and ZW conceived and designed the project. CZ, YS, JM, GL, and HW collected and organized the data. CZ, HW, GL, and XZ analyzed and interpreted the data. JS, RZ, ZL, ZW, DL, and XZ provided the technology and resources. All authors contributed to the article and approved the submitted version.

## Conflict of Interest

CZ, JM, YS, JS, RZ, ZW, and XZ were employed by the company Wens Foodstuff Group Co., Ltd. The remaining authors declare that the research was conducted in the absence of any commercial or financial relationships that could be construed as a potential conflict of interest.
