# Supplementary material for: Generation of Multi-Transgenic Pigs Using PiggyBac Transposons Co-expressing Pectinase, Xylanase, Cellulase, β-1.3-1.4-Glucanase and Phytase
Source: Front Genet. 2020 Nov 30;11:597841. doi: 10.3389/fgene.2020.597841 (PMC7734351; doi:10.3389/fgene.2020.597841)
Supplement: Supplementary file 1 [file Presentation_1.zip › Supplementary Figure 1 - 7 & Tables 1 - 6.doc]

***Supplementary Material***

**Supplementary Table 1. Primers used in vector construction.**

| Item | Strand | Sequences (5'→3') | Product size (bp) |
| --- | --- | --- | --- |
| PXAT | F | ATTTGTCAGGGCGCGCCAACTTAAGCTGCCACCATGGTGCAG | 4,653 |
|  | R | GAATTGATCGGCGCGCCAGCGCTCTCAGTAGCCGTTGGCCTTCAG |  |
| pspA | F | ACGGCTACTGAGAGCGCTGTAGTCTCCACACCTGTTACCACCATC | 3,063 |
|  | R | GATCGGCGCGCCAGCGCTTGGGATATTGAGATGCTTCAGATTG |  |

**Supplementary Table 2.** Primers used in PCR and probes in southern blotting.

| Genomic segment | Strand | Sequences (5'→3') | Product size (bp) |
| --- | --- | --- | --- |
| *mPSP* | F | GAGACTCCCAAGCACTCTTAGGCAC | 1,688 |
|  | R | GCCCAATAAAGAAGCACACAATAGC |  |
| *Pg7fn* | F | AGAAGGTGTGAAGTTTTGCTGA | 1,137 |
|  | R | AAGGTAATATTGGTGCCTGAGT |  |
| *XynB* | F | GCTCCGACAACACCGTGAAGACAGT | 1,875 |
|  | R | CATCCTGGTTCTGGCATTCCCTGAG |  |
| *EsAPPA* | F | AAAGCGTCGTCATCGTCTCCC | 1,124 |
|  | R | CACATCCCCTGAGCATTGCGTTC |  |
| *TeEGI* | F | GAGAAACAGGACGAATCTTGTAGCC | 1,178 |
|  | R | TCCTCCGTTTCCGACCTGTCCGTA |  |
| *β-actin* | F | CACTGCCGCATCCTCT | 628 |
|  | R | CTCCTGCTTGCTGATCCACATC |  |
| Probe | F | GCCCTGGTCTATGCCTTTATCCTCCTC | 1,317 |
|  | R | GCCAGGAAACTTGGTAAGGTTGAGCG |  |

**Supplementary Table 3**. Primers used in reverse transcriptase PCR (RT-PCR) and quantitative Real-time PCR (qPCR). RT-β-actin is a primer across intron.

| Item | Strand | Sequences (5'→3') | Product size (bp) |
| --- | --- | --- | --- |
| q-Pg7fn | F | GACGCCACCTCTGCTATCTC | 165 |
|  | R | AGGTCCTTCCCACTCCTTGT |  |
| q-XynB | F | GTGACCACAAGCAACCACTTTAAC | 111 |
|  | R | GGAGGATGAGCCTGAGGACTG |  |
| q-EsAPPA | F | AAAGCGTCGTCATCGTCTCC | 215 |
|  | R | GGACTGTGGGCATCCTTTCTTAG |  |
| q-TeEGI | F | AAGAACGCCGACTCCAACTAC | 178 |
|  | R | TAGCCCTCCACAGCCAGATAG |  |
| q-β-actin | F | CCACGAGACCACCTTCAACTC | 130 |
|  | R | TGATCTCCTTCTGCATCCTGT |  |
| RT-β-actin | F | CACTGCCGCGTCCTCCTCCT | 628/400 |
|  | R | CTCCTGCTTGCTGATCCACATC |  |

**Supplementary Table 4. Customized primary antibody information in western blotting.**

| Antibody | Host species | Epitope | Product size | Dilution |
| --- | --- | --- | --- | --- |
| XynB | Rabbit | CESYGDYNPGSGGTY | 24 kDa | 1: 1000 |
| TeEGI | Rabbit | NNPPSHEQHRAASC | 50 kDa | 1: 1000 |

**Supplementary Table 5.** Pig growth and fattening stage feed formulas. 1. Provided by Wenshi Food Group Co., Ltd., provided for each kg of ration (feeding basis): Vitamin A 6,500 IU, Vitamin D3 2,000 IU, Vitamin E 42.00 mg, Vitamin K3 2.00 mg, Vitamin B1 2.00 mg, Vitamin B2 6.40 mg, vitamin B6 3.00 mg, vitamin B12 0.02 mg, D-biotin 0.16 mg, D-pantothenic acid 20.00 mg, folic acid 1.20 mg, niacin 24.00 mg, Fe 159.00 mg, Zn 161.00 mg, Cu 142.00 mg, Mg 40.00 mg, I 0.40 mg, Se 0.30 mg, Co 0.10 mg, enramycin (8%) 100 mg, Antioxidant (Niuet) 85 mg. 2. Provided by Wenshi Food Group Co., Ltd., provided for each kg of diet (feeding basis): vitamin A 6,500 IU, vitamin D 2,000 IU, Vitamin E 40 mg, vitamin K3 2.00 mg, vitamin B1 2.00 mg, vitamin B2 5.00 mg, Vitamin B2 6.40 mg, vitamin B6 3.00 mg, vitamin B12 0.02 mg, biotin 0.15 mg, D-pantothenic acid 20.00 mg, folic acid 1.00 mg, niacin 24.00 mg, Fe 134.00 mg, Zn 125.00 mg, Cu 30.00 mg , Mg 50.00 mg, I 0.50 mg, Se 0.35 mg, Co 0.16 mg; Enramycin (8%) 100 mg. Antioxidant (Niuet) 85 mg.

| Component | Growth and fattening stage | | |
| --- | --- | --- | --- |
|  | 30~60 kg | 60~90 kg | 90~100 kg |
| Corn, level 1 | 361.5 | 260 | 240 |
| Wheat, level 2 | 235 | 311 | 220 |
| Barley | 129 | 175 | 200 |
| Rice bran | 50 | 32 |  |
| Wheat bran |  | 20 | 162 |
| Puffed soy flour | 149 | 135 | 96 |
| Soybean meal, 46% | 15 |  |  |
| Soybean oil | 20 | 31 | 50 |
| Stone powder | 16.6 | 12 | 11 |
| Premix^1^ | 10 |  |  |
| Premix^2^ |  | 10 | 10 |
| Lysine, 70% | 5.3 | 5.6 | 3.5 |
| Threonine, 98.5% | 2.1 | 1.7 | 0.9 |
| L-methionine, 98% | 1 | 0.8 | 0.4 |
| Tryptophan, 98% | 0.5 | 0.4 | 0.2 |
| Salt | 3 | 3 | 3 |
| Choline chloride, 60% | 1 | 1.5 | 2 |
| Mold inhibitor | 1 | 1 | 1 |
| Total, kg | 1,000 | 1,000 | 1,000 |
| Pig digestion energy, Kcal/kg | 3,339 | 3,294 | 3,209 |
| Crude protein, % | 14.94 | 13.9 | 12.96 |
| Total calcium, % | 0.69 | 0.62 | 0.63 |
| Total phosphorus, % | 0.4 | 0.38 | 0.39 |
| Digestible phosphorus, % | 0.103 | 0.124 | 0.153 |

**Supplementary Table 6.** Embryo transfer data for cloned pigs. TG pigs represents transgenic pigs.

| Pooled | Embryos | Transfers | Pregnant | Births | TG pigs |
| --- | --- | --- | --- | --- | --- |
| PXAT | 209 | 1 | return | - | - |
|  | 220 | 1 | return | - | - |
|  | 201 | 1 | normal | 3 piglets  (♂, 1 dead at 1 month) | 2 live and 1 dead |
|  | 213 | 1 | return | - | - |
|  | 221 | 1 | normal | 1 live piglet (♂) | - |
|  | 217 | 1 | return | - | - |
|  | 196 | 1 | normal | 3 piglets (2 live and 1 dead) | 1 dead |
|  | 184 | 1 | return | - | - |
|  | 215 | 1 | return | - | - |
|  | 220 | 1 | normal | 2 live piglets (♂) | 1 live |
| Total | 2,096 | 10 | 4 sows | 9 piglets (7 live and 2 dead) | 3 live and 2 dead |

**Supplementary Table 7.** Serum biochemical information for F1 transgenic pigs. These indicators were detected: Total protein (TP), albumin (ALB), globulin (GLOB), white ball ratio (A/G), glucose (GLU), creatinine (CRE), blood urea nitrogen (BUN), total bilirubin (TBIL), creatine kinase (CK), γ-glutamyl transferase (γ-GT), phosphorus (P), calcium (Ca), potassium (K+), sodium (Na+), chloride (CL-). Asterisk represents significant differences (t-test), wild type (WT): n = 3, transgenic (TG): n = 3.

| Ingredient | WT | TG | *P* values |
| --- | --- | --- | --- |
| TP (g/L) | 62.27 ± 1.48 | 62.75 ± 2.69 | 0.8931 |
| ALB (g/L) | 31.37 ± 1.36 | 32.5 ± 1.83 | 0.6625 |
| GLOB (g/L) | 30.9 ± 0.55 | 30.25 ± 0.89 | 0.5947 |
| A/G | 1.01 ± 0.05 | 1.07 ± 0.03 | 0.3379 |
| GLU (mmol/L) | 5.08 ± 0.42 | 3.31 ± 0.29^*^ | 0.0156 |
| CRE (μmol/L) | 140.27 ± 5.54 | 133.4 ± 4.4 | 0.3700 |
| BUN (mmol/L) | 1.96 ± 0.09 | 1.98 ± 0.01 | 0.8443 |
| TBIL (μmol/L) | 0.18 ± 0.06 | 0.33 ± 0.07 | 0.1887 |
| CK (U/L) | 863.67 ± 235.32 | 1,280.75 ± 342.41 | 0.3970 |
| γ-GT (U/L) | 60 ± 4 | 68.25 ± 5.45 | 0.3078 |
| P (mmol/L) | 2.79 ± 0.1 | 3.32 ± 0.11^*^ | 0.0223 |
| Ca (mmol/L) | 2.9 ± 0.02 | 2.88 ± 0.03 | 0.6375 |
| K+ (mmol/L) | 5.09 ± 0.27 | 5.12 ± 0.08 | 0.9332 |
| Na+ (mmol/L) | 141.03 ± 3.09 | 145.03 ± 0.3 | 0.1863 |
| CL- (mmol/L) | 104.1 ± 1.72 | 103.48 ± 1.51 | 0.7960 |

**Supplementary Figure 1.** Relative mRNA levels of *PXAT* in various tissues from 10-month old transgenic pigs. (**A**) mRNA levels of *Pg7fn* in various tissues. (**B**) mRNA levels of *XynB* in various tissues. (**C**) mRNA levels of *EsAPPA* in various tissues. (**D**) mRNA levels of *TeEGI* in various tissues. WT represents the parotid tissue of wild- type pig. Data is shown as mean ± SEM, n = 3.

**Supplementary Figure 2.** Reverse transcriptase PCR in various tissues from 10-month old transgenic pigs. M and W represent DNA marker and water; P and N indicate the DNA from positive and negative cell, respectively.

**Supplementary Figure 3.** F1 transgenic genotype identification. M and W represent DNA marker and water; P and N indicate the DNA from 920307 and wild-type pig, respectively. W1 and W2 represent 2 wild-type gilts, respectively.

**Supplementary Figure 4.** The enzyme activities of the 10-month old F1 transgenic pigs. WT and TG represent wild-type and transgenic pigs, respectively. Data is shown as mean ± SEM, n = 3 (t-test). * *P* < 0.05 or ** *P* < 0.01.

**Supplementary Figure 5.** Growth performance of F1 transgenic pigs and wild-type littermates during the growing period (30 kg to 100 kg). (**A**) Comparison of average daily weight gain. (**B**) Comparison of average daily feed intake. (**C**) Comparison of feed/gain ratio. (**D**) Comparison of feeding time. Multivariate analysis of variance (MANOVA) was performed using the GLM procedure, with sex and initial weight used as the covariate. Data is expressed as mean ± SEM. WT and TG represent wild-type and transgenic pigs, respectively. *P* < 0.1 was used to describe trends.

**Supplementary Figure 6.** Growth curve of F1 transgenic pigs (30 kg to 100 kg). The blue and orange lines represent the growth curves of wild-type and transgenic pigs, respectively. The dark gray line represents the standard deviation. d: day.

**Supplementary Figure 7.** Comparison of enzyme activity between transgenic pigs (TG) and cattle. The samples were derived from the saliva of 18-month-old pigs and the rumen fluid of 4-year-old intubated cows. Data is shown as mean ± SEM, n = 3 (t-test). * *P* < 0.05 or ** *P* < 0.01.
